# Dimensions of Delusions and Attribution Biases along the Continuum of Psychosis

**DOI:** 10.1371/journal.pone.0144558

**Published:** 2015-12-07

**Authors:** Suzanne Ho-wai So, Venus Tang, Patrick Wing-leung Leung

**Affiliations:** 1 Department of Psychology, The Chinese University of Hong Kong, New Territories, Hong Kong Special Administrative Region, China; 2 Department of Clinical Psychology, Prince of Wales Hospital, Shatin, Hong Kong Special Administrative Region, China; IIBB/CSIC/IDIBAPS, SPAIN

## Abstract

This study compared delusional dimensions and attribution biases along the continuum of psychosis. Participants completed questionnaires on delusion-like beliefs and attributions. Although patients with first-episode psychosis (*N* = 70) endorsed fewer delusion-like beliefs than non-clinical individuals with psychotic-like experiences (*N* = 12), they scored highest on delusional conviction, distress and preoccupation, followed by non-clinical individuals with psychotic-like experiences, and then healthy controls (*N* = 642). Self-serving bias was found in patients and non-clinical individuals with psychotic-like experiences, but not in healthy controls. Personalizing bias for negative events was not significantly different across the three groups. When compared with healthy controls, non-clinical individuals with psychotic-like experiences had an exaggerated self-serving bias, but were not more marked in personalizing bias. Self-serving bias and personalizing bias were both associated with delusional dimensions. However, the association between self-serving bias and number of delusion-like beliefs was stronger among patients than non-clinical participants. Future research could investigate the extent to which self-serving bias, in combination with an appraisal of delusional ideation as convincing, distress, and preoccupying, contributes to the development of clinical delusions.

## Introduction

The view of psychosis as a continuum has been supported by two main lines of research—that psychotic symptoms are reported across clinical and non-clinical populations, and that psychosocial factors that predict the development of psychotic symptoms in patients also exert similar impacts in non-clinical individuals [1–8].

Studies assessing delusions multi-dimensionally found that dimensions/ aspects of delusional experience, especially distress and preoccupation, are more important than number of delusions in distinguishing patients from community samples [9–11]. However, these studies did not distinguish, within their community samples, those who met the criteria for psychosis from those who did not have psychotic experiences. Therefore, the present study aims at extending the literature and investigates the differences in delusional dimensions between the clinical group (i.e., clinical patients with psychosis) and those in the community who have a high risk of psychosis, but who have not sought treatment. We would expect that clinical patients with psychosis will score higher on delusional dimensions (conviction, distress and preoccupation) than individuals in the community who may have a high risk of psychosis, but are not seeking treatment.

Previous research has shown that patients with delusions tend to attribute positive events to the self and negative events to external causes (i.e., self-serving bias, ‘SSB’), especially to other people (i.e., personalizing bias, ‘PB’) [12–15], with inconsistent associations to delusions and their subtypes [16–19]. Psychological treatments aiming at treating delusions via modifying cognitive biases, including attributions, have been developed and tested (see review by [20]). However, less is known about which aspect(s) of delusions are linked with SSB/PB, and how the biases compare between patients and non-help-seeking individuals with a high risk of psychosis. Janssen et al. [21] found that individuals with subclinical psychotic experiences (above 75^th^ percentile) did not differ from healthy controls in personalizing bias. Using a screening instrument to identify community individuals with a high risk of psychosis, this study hypothesizes that there will be stronger SSB and PB in clinical patients than in non-clinical individuals and explores the association between attributions and delusionality across groups.

## Materials and Methods

### Participants

Research ethics was approved by the Kowloon West Cluster Clinical Research Ethics Committee in Hong Kong. Chinese outpatients with a case note diagnosis of schizophrenia spectrum disorder according to the Diagnostic and Statistical Manual of Mental Disorders [22] were recruited from a service for first episode psychosis (FEP) in Hong Kong. Patients with a primary diagnosis of substance use disorder, mood disorder, organic mental disorder or intellectual disability were excluded.

A total of 70 patients with FEP and 654 non-clinical, community participants were recruited to this study. Formal psychiatric diagnosis was available in 38 clinical patients (31: Schizophrenia spectrum disorder; 7: Schizophrenia). On average, they had been receiving psychiatric service for 20.2 months (range: 0–48). The clinical and non-clinical participants were matched on the key demographic variables. Independent-sample *t* tests and chi-square tests revealed no significant group difference (*p* > .05) in age (Clinical: *M =* 20.01, *SD* = 3.31; Non-clinical: *M =* 20.98, *SD* = 4.10), years of education (Clinical: *M =* 10.79, *SD* = 2.32; Non-clinical: *M =* 10.97, *SD* = 1.96), and gender (Clinical: 47.1% male; Non-clinical: 41.6% male).

Non-clinical, community individuals were recruited from schools and universities, community organizations, and the Internet. Exclusion criteria were as follows: Personal or family history of mental illness, current or previous substance use, history of brain injury or organic mental disorders.

### Measures and Procedure

Upon written consent, participants completed the following self-reported questionnaires individually, with researcher’s assistance when required.

#### Attribution style

On the Internal, Personal and Situational Attributions Questionnaire (IPSAQ; [23]), participants were asked to categorize the causes of 16 positive and 16 negative situations as internal, external-personal or external-situational.

The SSB score was obtained by subtracting the percentage of internal attributions for negative events from the percentage of internal attributions for positive events, with an SSB score >0 indicating the presence of SSB.

The PB score is the number of external-personal attributions for negative events divided by the total number of external-personal and external-situational attributions for negative events. A PB score of 0.5 or above indicates the presence of PB [23].

#### Delusional ideation

The Peters et al. Delusions Inventory (PDI; [24]) consists of 21 delusion-like beliefs. For each belief endorsed, participants were asked to rate on their levels of conviction, distress, and preoccupation. The PDI has high internal consistency (0.88) and test-retest reliability (*r* = 0.82; [24]).

#### Psychotic symptom rating

The Community Assessment of Psychic Experiences (CAPE; [25]) is a questionnaire measuring positive (20 items), negative (14 items) and depressive symptoms (8 items) in the general population. For each symptom, participants are asked to report its frequency on a 4-point scale. For any item that is endorsed as present, participants are then asked to rate on the level of distress. Therefore, the frequency and distress scores range respectively from 20–80 for the positive subscale, 14–56 for the negative subscale, and 8–32 for the depressive subscale.

Cutoff scores of the positive subscale have been reported for reliable detection of ultra-high risk for psychosis [26]. According to Boonstra, Wunderink, Sytema, and Wiersma [27], a positive frequency score of 50 detected FEP with sensitivity of 77.5 and specificity of 70.5. Therefore, the present study divided non-clinical participants into two subgroups according to this criterion—participants who had CAPE positive frequency score above 50 were categorized as “non-clinical individuals with psychotic-like experiences (NC-P)”, whereas those who scored at or below 50 on the CAPE positive frequency score were categorized as “non-clinical individuals with a low risk of psychosis (NC-NP)”.

All participants completed IPSAQ and PDI, whereas CAPE was completed by non-clinical participants only.

### Statistical Analysis

Statistical analyses were conducted using IBM SPSS Statistics for Windows, Version 19.0 [28]. Delusional dimensions and attributional biases were compared across three groups using one-way ANOVA and post-hoc Bonferroni tests. The ANOVA main results were corrected for the number of scales used.

Relationship between attributional biases (SSB and PB) and delusional dimensions was analyzed using linear regression models (forced entry method). The first set of models, using data from the whole sample (*N* = 724), included both SSB and PB as IVs, and the following PDI scores as DVs respectively: number of beliefs, mean level of conviction, mean level of distress, and mean level of preoccupation. Significance level of regression analysis was corrected for the number of models tested. Where SSB or PB showed a significant prediction of a PDI score, a second set of regression models were tested, which included Group, Group x SSB and Group x PB interaction effects as IVs. This was to ascertain whether the predictive effect of the attributional biases differs between clinical (*N* = 70) and non-clinical (*N =* 654) individuals.

## Results

### Group Comparison of Delusion Dimensions

Using the Boonstra et al. [27] cutoff criterion for psychosis, 12 participants (1.8%) scored > 50 on the CAPE positive frequency score and were categorized as ‘non-clinical individuals with psychotic-like experiences’ (NC-P) group, whereas 642 (98.2%) were categorized as ‘non-clinical individuals with a low risk of psychosis’ (NC-NP).

The PDI scores across groups are shown in Table 1. One-way ANOVA and post-hoc Bonferroni tests revealed that, across the three delusional dimensions (i.e. conviction, distress and preoccupation), the FEP group scored higher than the NC-P group, whose score was in turn higher than that of the NC-NP group. The overall group differences remained significant after controlling for the four scales used (i.e. *p* < .0125).

**Table 1:** Group comparisons of delusional ideation and attributional style.

	FEP Group	NC-P Group	NC-NP Group	ANOVA	Bonferroni	Bonferroni	Bonferroni
	(*n* = 70)	(*n* = 12)	(*n* = 642)		FEP vs. NC-P	NC-P vs. NC-NP	FEP vs. NC-NP
	M (*SD*)	M (*SD*)	M (*SD*)				
					*MD* = 1.46	*MD* = 0.76	*MD* = 2.21
**Conviction**	3.01	1.55	0.79	*F* (2,721) = 288.68	*SE* = 0.23	*SE* = 0.21	*SE* = 0.09
	(1.15)	(0.29)	(0.68)	*p* < .001	*p* < .001	*p* = .001	*p* < .001
					*MD* = 1.37	*MD* = 0.66	*MD* = 2.03
**Distress**	2.94	1.57	0.91	*F* (2,721) = 191.58	*SE* = 0.26	*SE* = 0.24	*SE* = 0.10
	(1.06)	(0.29)	(0.81)	*p* < .001	*p* < .001	*p* < .05	*p* < .001
					*MD* = 1.21	*MD* = 0.74	*MD* = 1.95
**Preoccu- pation**	2.76	1.54	0.80	*F* (2,721) = 227.15	*SE* = 0.23	*SE* = 0.21	*SE* = 0.09
	(1.12)	(0.25)	(0.68)	*p* < .001	*p <* .001	*p <* .01	*p <* .001
					*MD* = -7.95	*MD* = 13.67	*MD* = 5.72
**Number of beliefs**	6.97	14.92	1.25	*F* (2,721) = 386.20	*SE* = 0.72	*SE* = 0.67	*SE* = 0.29
	(5.62)	(3.34)	(1.54)	*p* < .001	*p* < .001	*p* < .001	*p* < .001
					*MD* = -7.76	*MD* = 17.36	*MD =* 9.60
**Self-serving bias**	6.27	14.02	-3.34	*F* (2,721) = 13.70	*SE =* 5.66	*SE* = 5.28	*SE* = 2.28
	(16.14)	(14.01)	(18.39)	*p* < .001	*p* = .513	*p* < .01	*p* < .001
					*MD* = 0.09	*MD* = -0.03	*MD* = 0.06
**Personal-izing bias**	0.80	0.71	0.74	*F* (2,720) = 3.20	*SE =* 0.06	*SE* = 0.06	*SE =* 0.03
	(0.22)	(0.09)	(0.20)	*p* = .042	*p* = .402	*p* = 1.000	*p* = .044

*Notes*: Levels of conviction, distress and preoccupation for each endorsed delusion-like belief were measured using the PDI [14, 24]. The number of beliefs indicates the mean number of delusion-like beliefs endorsed on the PDI.

NC-P endorsed the greatest number of delusion-like beliefs, followed by FEP, and then NC-NP.

Within the clinical group, there was no significant correlation between duration of psychiatric service received and any of the PDI scores (*p* > .05).

### Group Comparison of Attributional Biases

As shown in Table 1, there was a weaker SSB tendency in NC-NP than both FEP (*d* [95% CI] = -0.53 [-0.78, -0.28]) and NC-P (*d* [95% CI] = -0.95 [-1.52, -0.37]). The difference between the NC-P and FEP groups did not reach statistical significance (*d* [95% CI] = 0.49 [-0.13, 1.10]). The NC-NP group also had a significantly lower proportion of individuals showing an SSB (35.8%) than both the FEP (62.9%, χ^2^(1, *N* = 82) = 19.48, *p* < .001) and NC-P (91.7%, χ ^2^(1, *N* = 654), *p* < .001) groups.

There was a significant group difference in PB. However, after correcting for the number of comparisons (i.e. 2), the overall group difference no longer remained significant (*p* > .025). PB for negative events was stronger in FEP than NC-NP group (*d* [95% CI] *=* 0.30 [0.05, 0.55]). The difference between the FEP and NC-P groups (*d* [95% CI] = 0.44 [-0.19, 1.05]) and that between the NC-P and NC-NP groups (*d* [95% CI] = -0.15 [-0.72, 0.42]) did not reach statistical significance.

### Association between Attributional Biases and Delusional Dimensions

**Fig 1:**
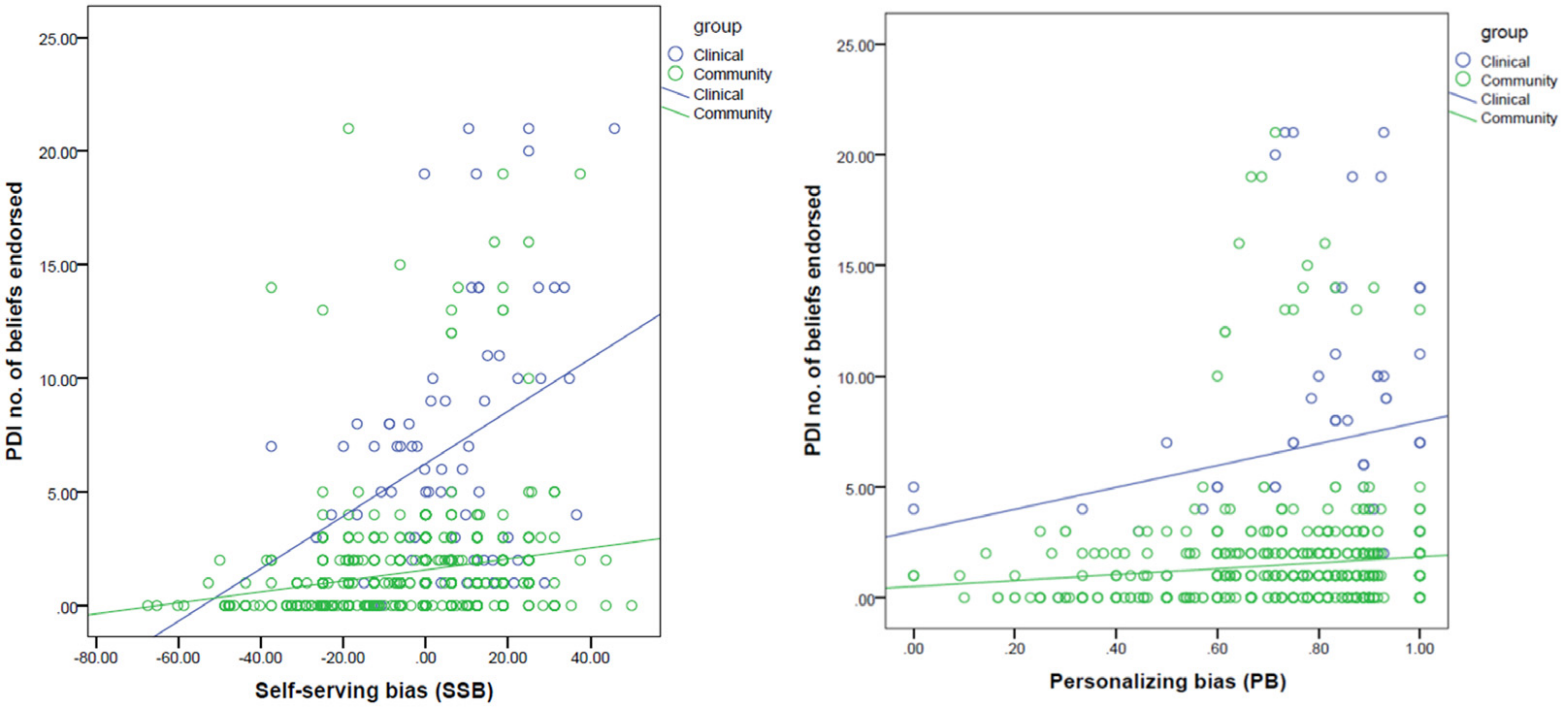
Association between attributional biases and number of delusional ideations.

Linear regression models with SSB and PB as independent variables and PDI number of beliefs and three dimensions as respective dependent variables (*N =* 724) reached statistical significance (*p* < .05). PDI number of beliefs was positively associated with both SSB (Beta = 0.241, *t* = 6.730, *p* < .001) and PB (Beta = 0.141, *t* = 3.935, *p* < .001), which remained significant after correcting for the number of tests. The Group x SSB interaction effect was significant (Beta = -1.050, *t* = -4.244, *p* < .001), with a stronger association between number of beliefs and SSB in patients than in healthy individuals (Fig 1).

**Fig 2:**
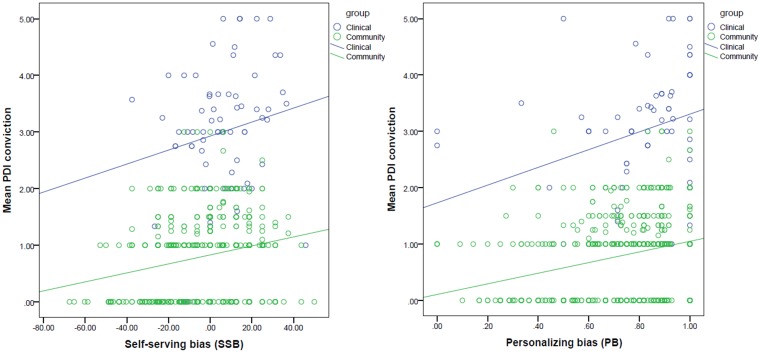
Association between attributional biases and level of delusional conviction.

Level of conviction was positively associated with both SSB (Beta = 0.247, *t* = 7.118, *p* < .001) and PB (Beta = 0.257, *t* = 7.399, *p* < .001), which remained significant after correcting for the number of tests. The association between conviction and either bias was not significantly different between groups (*p* levels of interaction effects > .05; Fig 2).

**Fig 3:**
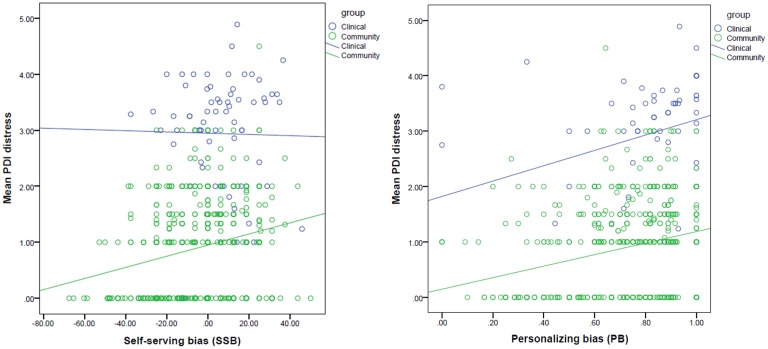
Association between attributional biases and level of delusional distress.

Level of distress was positively associated with both SSB (Beta = 0.243, *t* = 6.994, *p* < .001) and PB (Beta = 0.254, *t* = 7.307, *p* < .001), which remained significant after correcting for the number of tests. The Group x SSB interaction effect fell out of the corrected significance level (Beta = 0.460, *t* = 2.027, *p* = .043; Fig 3).

**Fig 4:**
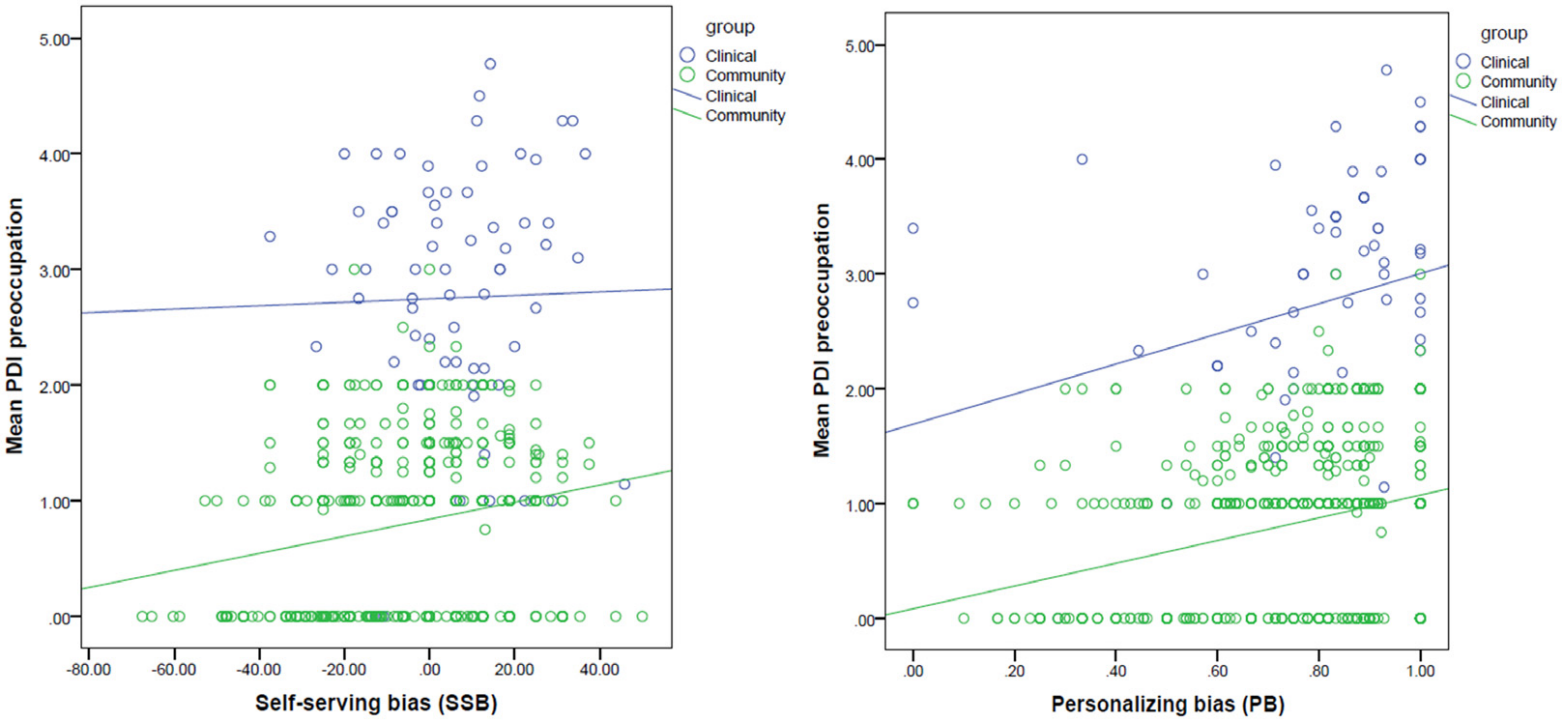
Association between attributional biases and level of delusional preoccupation.

Level of preoccupation was positively associated with both SSB (Beta = 0. 222, *t* = 6.367, *p* < .001) and PB (Beta = 0.268, *t* = 7.682, *p* < .001), which remained significant after correcting for the number of tests. The association between preoccupation and either bias was not significantly different between groups (*p* levels of interaction effects > .05; Fig 4).

## Discussion

This study compared delusional dimensions and attributional biases between clinical patients with first-episode psychosis and non-clinical individuals, who were subdivided according to a self-report screening tool. It provided identification of a subgroup of community individuals who experienced psychotic symptoms but were not seeking formal clinical care.

This study supported the hypothesis of a graded difference in delusional dimensions among patients with first-episode psychosis, individuals in the community with psychotic-like experiences, and healthy individuals. Patients reported fewer delusional beliefs than non-clinical individuals with risk of psychosis, but were more convinced, distressed and preoccupied with their delusional experiences. Our data adds to the evidence that aspects/dimensions of the delusional experience are more associated with patient status than the absolute number of delusion-like beliefs [9, 11]. Our data also supports the argument that it is the appraisal of anomalous experiences, rather than the experiences per se, that defines the psychopathology of delusions [29].

Consistent with Janssen et al. [21], we found that non-clinical individuals with psychotic-like experiences did not show significantly stronger personalizing bias (PB) than healthy controls. Although the clinical group reported stronger PB than the non-clinical non-psychotic group, as hypothesized, the effect size was modest. Therefore, PB did not differentiate the three groups varying on delusionality. It is of note that previous studies that reported a group difference on PB mostly focused on persecutory delusions, and there is evidence that PB is more consistently associated with the theme of persecution or hostility than with the presence of delusional ideation on the whole [30–32]. As we did not limit our sample to individuals with persecutory delusions, our data did not allow a sensitive test of the graded difference in PB across groups. On the other hand, non-clinical individuals with psychotic-like experiences displayed an exaggerated self-serving bias (SSB) than healthy controls, and were not significantly different from patients. More importantly, although both SSB and PB were associated with delusional severity and dimensions, the association between SSB and number of delusion-like beliefs was stronger among patients than non-clinical individuals.

Altogether, our results suggested the possibility that SSB is more closely linked to the presence of delusional ideation than PB, and that SSB has a stronger impact on number of delusional beliefs when delusional beliefs are of clinical intensity (i.e. a dose-response relationship). On the other hand, SSB alone did not contribute to clinical delusions leading to need for care. Rather, both SSB and an evaluation of the delusional ideation as convincing, distressing and preoccupying distinguished patients from the high-risk group. The suggestion that SSB, in combination with delusional dimensions, contributes to the development of clinical delusions is a hypothesis that needs to be considered with caution because our study was limited by a cross-sectional design and use of self-reported measures of delusionality and attributions. It is also noteworthy that our healthy group reported a weaker SSB than previous studies. Although this is consistent with the view that SSB is more robust in the Western cultures and is smaller in Asian samples [33–35], we do not consider our finding conclusive given a relatively wide range of SSB scores in our sample.

One of the limitations of this study was the small group of non-help-seeking individuals with a high risk of psychosis (*N* = 12). The small size of this group limited the power of group comparisons, especially for differences that had a moderate effect size but did not reach statistical significance due to the small sample size. Whilst we identified non-clinical individuals with psychotic-like experiences according to an established psychometric criterion using the CAPE, a more reliable screening method would be a comprehensive diagnostic interview. Moreover, the CAPE cut-off score for psychotic-like experiences used in this study was developed based on help-seeking clinical samples. Since there is a much lower base rate of psychosis in the community compared to the clinics, we cannot assume that the sensitivity and specificity of the scale would be the same in the community. In addition, we did not have the patients’ complete symptom profile. This limits the generalizability of our results in general, and makes it harder to interpret the PB results in particular, because PB has previously been linked more specifically to persecutory delusions than other subtypes of delusions. This study also lacked comprehensive assessment of emotional distress, which has been suggested to contribute to the development of delusions [36–38]. Therefore, future research on the relationship between risk of delusions and attributional biases and the underlying mechanisms is warranted, with more balanced groups of individuals representing various points along the psychosis continuum, and improved methodology of assessment.

## Supporting Information

S1 File Dataset for present study. (XLSX)
